# Medical prediction from missing data with max-minus negative regularized dropout

**DOI:** 10.3389/fnins.2023.1221970

**Published:** 2023-07-13

**Authors:** Lvhui Hu, Xiaoen Cheng, Chuanbiao Wen, Yulan Ren

**Affiliations:** ^1^School of Intelligent Medicine, Chengdu University of Traditional Chinese Medicine, Chengdu, China; ^2^Sinology College of Chengdu University of Traditional Chinese Medicine, Chengdu, China

**Keywords:** medical prediction, missing data, dropout, regularization, negative sampling

## Abstract

Missing data is a naturally common problem faced in medical research. Imputation is a widely used technique to alleviate this problem. Unfortunately, the inherent uncertainty of imputation would make the model overfit the observed data distribution, which has a negative impact on the model generalization performance. R-Drop is a powerful technique to regularize the training of deep neural networks. However, it fails to differentiate the positive and negative samples, which prevents the model from learning robust representations. To handle this problem, we propose a novel negative regularization enhanced R-Drop scheme to boost performance and generalization ability, particularly in the context of missing data. The negative regularization enhanced R-Drop additionally forces the output distributions of positive and negative samples to be inconsistent with each other. Especially, we design a new max-minus negative sampling technique that uses the maximum in-batch values to minus the mini-batch to yield the negative samples to provide sufficient diversity for the model. We test the resulting max-minus negative regularized dropout method on three real-world medical prediction datasets, including both missing and complete cases, to show the effectiveness of the proposed method.

## 1. Introduction

With the rapid development of deep learning techniques, deep neural networks have gained popularity as a tool for solving complex problems in various domains. Due to its potential to increase healthcare's accuracy and effectiveness, the use of deep learning in medical prediction has drawn a lot of attention (Miotto et al., 2018; Ayon and Islam, 2019). The analysis and prediction of medical data is a challenging issue because most of the medical data is incomplete in nature.

Missing/incomplete data is a pervasive problem in medical research, arising from various reasons, including non-response to questionnaires, loss to follow-up, and data entry errors from institutions (Waljee et al., 2013; Kumar et al., 2017). However, for deep learning methods, high data quality standards are crucial to ensure robust predictive performance, and missing data can lead to biased estimation (Jakobsen et al., 2017). Therefore, there is a need for model development to be more robust to keep a high accuracy in the presence of missing data (Bell et al., 2014; Mehrabani-Zeinabad et al., 2020).

Typically, there are a variety of approaches to handling the missing data cases. List-wise deletion (King et al., 1998), where any missing value in their categorical variables are completely excluded from the data, is the simplest way. However, it causes a loss of information and brings big problems in many missing data situations. Another approach to address missing data is imputation (Schafer and Graham, 2002); in this method, the missing data is replaced with plausible substitutions based on the observed data (Batista and Monard, 2003; Mazumder et al., 2010). Unfortunately, due to the inherent uncertainty of imputation, some missing values might be incorrectly imputed, which is considered noise. This would make the model overfit the observed data distribution when learning from the noise data and have a negative impact on the model generalization performance.

Regularization is a good way to reduce overfitting in neural networks by discouraging learning a more complex or flexible model. By randomly dropping out different subsets of neurons during training, the dropout (Srivastava et al., 2014) technique prevents the network from relying too heavily on any particular feature or combination of features, making it more robust to the noise imputation cases. Recently, R-Drop (Wu et al., 2021) is proposed to alleviate the inconsistency between the training and inference stages by forcing the output distributions of different sub-models generated by dropout to be consistent with each other, resulting in a better model generalization ability. Nevertheless, R-Drop often results in slower convergence and introduces some instability during training. Moreover, R-Drop does not consider the difference between positive and negative samples, which prevents the model from learning robust representations and inhibits generalization.

To handle the aforementioned problem, in this work, we propose a novel max-minus negative regularized dropout scheme to improve the model generalization ability, particularly in the context of missing data. Concretely, in each mini-batch training, we maintain the consistency between two distributions from the same data sample (positive) by different dropout sub-models like R-Drop. Additionally, we consider involving the inconsistency between the output distributions of positive and negative samples. Involving negative samples would make the model learn more robust representations and avoid overfitting. Previous studies (Schroff et al., 2015; Ge et al., 2021) elucidate that appropriately choosing the negative samples is a critical component in deep learning. We find that the traditional negative sampling strategy of directly treating different in-batch input data as negative samples are insufficient for imputation cases. We design a new max-minus negative sampling technique that uses the maximum in-batch values to minus the mini-batch to yield the negative samples providing sufficient diversity for the model.

The main contributions of this work are summarized as follows.

- We propose a simple, yet effective negative regularization scheme built upon R-Drop, which maintains both the consistency between two distributions from the same data sample and the inconsistency between the output distributions of positive and negative samples.
- We design a new max-minus negative sampling strategy, which facilitates convergence and is more effective compared to the traditional in-batch negative example sampling strategy.
- The resulting max-minus negative regularized dropout method can be easily applied to both complete and incomplete/missing data cases to boost model performance and generalization ability. Extensive experiments and ablation studies are performed on three real medical prediction datasets to demonstrate the effectiveness of the proposed method, particularly in the context of missing data.

The rest of the article is organized as follows: After summarizing related work in Section 2, we describe some preliminaries of Swin Transformer and R-Drop in Section 3. Then, we introduce the proposed method in Section 4. In Section 5, we conduct a series of experiments to verify the performance of the proposed method. Section 6 concludes this work.

## 2. Related work

In this section, we provide an overview of the relevant literature, focusing on the imputation of missing data, regularization techniques, and their applications in a variety of contexts.

### 2.1. Imputation for missing data

Missing data can significantly hinder the improvement of classification accuracy (Donders et al., 2006), especially in medical research, where missing values are common. To address this issue, imputation has become a common method for dealing with missing data (Graham et al., 2013), which mainly involves mean/mode imputation, multiple imputation, Bayesian imputation, and regression imputation techniques. Mean/mode imputation replaces missing data with the mean/mode of the available/observed data (Schneider, 2001; Thirukumaran and Sumathi, 2012). Multiple imputation entails generating multiple plausible imputations for each missing value and combining the results to produce a final estimate (Thirukumaran and Sumathi, 2012). Based on the observed data, Bayesian imputation generates multiple estimates for each missing value (Ma and Chen, 2018). Regression imputation predicts missing values based on other variables in the dataset using a regression model (Thirukumaran and Sumathi, 2012). Note that multiple imputation, Bayesian imputation, and regression imputation demand a significant amount of computational resources when applied to large datasets (Templ et al., 2011; Enders et al., 2016). For continuous data, one common approach is mean imputation and regression imputation (Musil et al., 2002; Zhang et al., 2022). Mode imputation, random imputation, and Bayesian imputation are commonly used to deal with the missing data with a boolean value (Bielza and Larrañaga, 2014; Miller et al., 2016).

### 2.2. Regularization methods

Deep neural networks are capable of learning complex patterns in data, which can be used for a wide range of applications (Amit, 2019; Spoon et al., 2021; Li et al., 2022; Yang et al., 2023). However, neural networks can be susceptible to overfitting, which occurs when a model is trained to match the training data too closely and consequently fails to adapt to new, unseen data. To address overfitting in deep models, numerous regularization techniques have been proposed, e.g., dropout (Gal and Ghahramani, 2016), weight decay (Loshchilov and Hutter, 2019), constraint (Teipel et al., 2017; Fan et al., 2018; Wong et al., 2018), etc. Among these methods, the dropout technique and its variants have gained popularity due to their effectiveness, moderate cost, and compatibility with other regularization methods in neural network architecture (Moradi et al., 2020; Pham and Le, 2021). Due to their ability to promote sparsity of weights and their stochastic nature, dropout methods have also been adapted for use in other applications, such as contrastive learning for sentence representation learning (Wang et al., 2018; Gao et al., 2021) and model uncertainty estimation (Gal and Ghahramani, 2016; Li and Gal, 2017).

In this paper, we test different imputation techniques for incomplete medical prediction data. Besides, we propose a simple, yet effective max-minus negative regularization method built upon R-Drop to improve the model generalization ability by involving negative samples in training. Unlike previous works, we design a new max-minus negative sampling strategy to obtain more semantically dissimilar negative samples, which would make the model learn more robust representations.

## 3. Background

### 3.1. Notation

Now we present some necessary notations in this paper. For the training dataset $D_{tr}={\left\{\left(x_{i},y_{i}\right)\right\}}_{i=1}^{n}$, *n* is the number of the samples, *x*_*i*_ and *y*_*i*_ are the input sample and corresponding label, respectively. (*x*_*i*_, *y*_*i*_) denotes the labeled data pair. For example, in medical treatment, *x*_*i*_ can be the clinical features, such as dizziness, course of treatment, etc., and *y*_*i*_ is the corresponding target disease. The goal of the model optimization is to learn a model prediction P(y|x,w). The probability distribution of the mapping function is also denoted as P(y|x,w), and the Kullback-Leibler (KL) divergence between two distributions $P^{1}$ and $P^{2}$ is represented by $D_{KL}\left(P^{1}||P^{2}\right)$.

For a classification task, given the training data $D_{tr}={\left\{\left(x_{i},y_{i}\right)\right\}}_{i=1}^{n}$, the main learning objective for a deep learning model is to minimize the cross-entropy loss function, which is as follows:


(1) $$\begin{array}{l} L_{CE}=-\frac{1}{n}\overset{n}{\underset{i}{\sum }}y_{i}\log\left(P\left(y_{i}|x_{i},w\right)\right). \end{array}$$

### 3.2. Swin Transformer

Swin Transformer (Liu et al., 2021), which is derived from Transformer (Vaswani et al., 2017), is an image classification model and has been widely used in numerous scenarios. It first adopts a hierarchical interval sampling strategy to gradually divide the image into many local images, and each local image produces a local feature by the Swin Transformer. The Swin Transformer uses the patch merging module with a hierarchical structure for reducing the resolution and adjusting the number of channels in the image. Furthermore, this design can save a certain amount of computational consumption. Swin Transformer contains two mechanisms: W-MSA and SW-MSA, which could perform multi-scale self-attention calculation on the input feature map and increase the receptive field of the model by window translation operation.

### 3.3. R-Drop

Although the introduction of dropout regularizes well in many scenarios, it may cause inconsistencies in the training and reasoning process. In order to alleviate this issue, R-Drop (Wu et al., 2021) proposes a simple consistency training technique, which forces the output distributions of various dropout-generated sub-models to be consistent with each other, to regularize dropout. Specifically, at each training step, R-Drop feeds the input data *x*_*i*_ to the network's forward pass twice to produce two distributions of the model predictions, denoted as $P^{1}\left(y_{i}|x_{i},w\right)$ and $P^{2}\left(y_{i}|x_{i},w\right)$. Since the dropout operator randomly drops units in a model, the distributions of $P^{1}\left(y_{i}|x_{i},w\right)$ and $P^{2}\left(y_{i}|x_{i},w\right)$ are different for the same input *x*_*i*_. Then, R-Drop regularizes the model predictions by minimizing the bidirectional KL divergence between $P^{1}\left(y_{i}|x_{i},w\right)$ and $P^{2}\left(y_{i}|x_{i},w\right)$, such that:


(2) $$\begin{array}{r} L_{KL}^{i}=\frac{1}{2}(D_{KL}\left(P^{1}\left(y_{i}|x_{i},w\right)\|P^{2}\left(y_{i}|x_{i},w\right)\right)+\\ D_{KL}\left(P^{2}\left(y_{i}|x_{i},w\right)\|P^{2}\left(y_{i}|x_{i},w\right)\right)). \end{array}$$

Making the trained model's error on the test set as small as feasible is one of the main goals of machine learning research. The test error rate of a full model, *R*(***f***_Full_), is defined as:


(3) $$\begin{array}{r} \begin{array}{r} R(f_{Full})\leq \underset{\epsilon_{gb}}{\underset{︸}{\left|R(E_{\varepsilon }(f_{\varepsilon }))-R_{T}(E_{\varepsilon }(f_{\varepsilon }))\right|}}+ \end{array}\\ \begin{array}{l} \underset{\epsilon_{te}}{\underset{︸}{R_{T}(E_{\varepsilon }(f_{\varepsilon }))}}+\underset{\epsilon_{sf}}{\underset{︸}{\left|R(f_{Full})-R(E_{\varepsilon }(f_{\varepsilon }))\right|}}, \end{array} \end{array}$$

where *R*_*T*_(*E*_ε_(*f*_ε_)) denotes the training error of sub-models, *R*(*E*_ε_(*f*_ε_)) denotes the test error sub-models, ϵ_*gb*_ is the generalization bound of the sub-models, ϵ_*te*_ denotes training error in the training process, and ϵ_*sf*_ indicates the gap between the sub-models and full model. R-Drop shows that the gap between the sub-model and full model is upper bounded by the gap between the sub-models:


(4) $$\begin{array}{l} \epsilon_{sf}\leq O\underset{\text{gap between sub-models}}{\underset{︸}{\left(E_{\varepsilon ,\varepsilon^{\prime },\varepsilon !=\varepsilon^{\prime }}\left(G\left(f_{\varepsilon^{\prime }}f_{\varepsilon^{\prime }}\right)\right)\right)}}. \end{array}$$

R-Drop optimizes the bidirectional KL-divergence of sub-models (shown in Equation 2) to alleviate the training-inference mismatch in the deep neural network model with a dropout mechanism.

## 4. Proposed method

**Figure 1:**
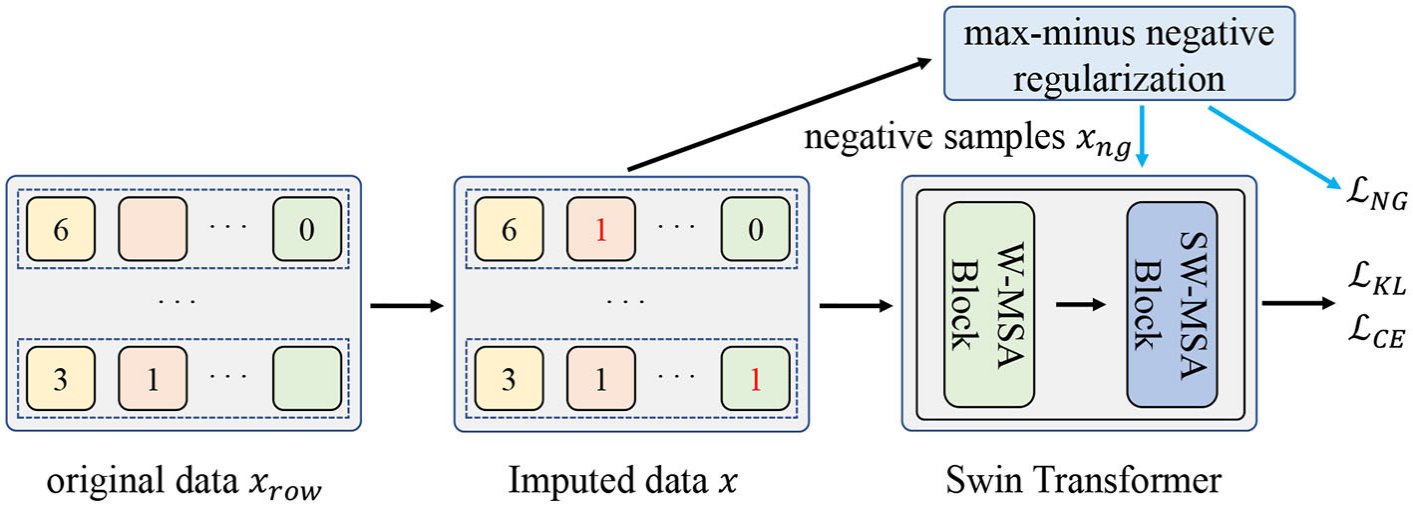
The overall flow diagram of the proposed method. Firstly, the original missing data *x*_*row*_ will be imputed as *x*. Then, the negative samples *x*_*ng*_ are generated from the proposed max-minus negative sampling technique. Finally, the input data *x* and negative samples *x*_*ng*_ are used to train the model. $L_{CE}$ is the loss function of the classification task. $L_{KL}$ and $L_{NG}$ are used to jointly improve the model generalization ability.

In this section, we first introduce the missing data imputation process. Then we present the negative regularization enhanced R-Drop scheme that involves the negative samples to further improve the model generalization and elaborate the proposed max-minus negative sampling technique. Finally, we give the pseudocode of the proposed method. Figure 1 shows the overall flow diagram of the proposed method.

### 4.1. Imputation for missing data

In medical research, missing data is a prevalent issue caused by a variety of factors, including loss to follow-up, incomplete responses to questionnaires or surveys, and data entry errors. If missing data cannot be handled properly, they can result in misleading estimations and have a negative impact on the model generalization performance. To avoid bias and improve the precision of their findings, it is essential that researchers select an appropriate method for handling missing data.

Imputation is a widely used technique for handling missing data cases. In medical research, some features are characterized by continuous values, such as the course of treatment and the age of the patient. While many other features, such as sex and dizziness, are represented by boolean values. Hence, the imputation operation is different for continuous and boolean values.

In this paper, we tried mode imputation, random imputation, and Bayes imputation to deal with the boolean missing data and employed the average imputation to impute the continuous missing data. We evaluate the effect of different imputation techniques through a variety of classification models. In light of their prediction results, the mode imputation technique is applied in our final implementation.

### 4.2. R-Drop with negative regularization

**Figure 2:**
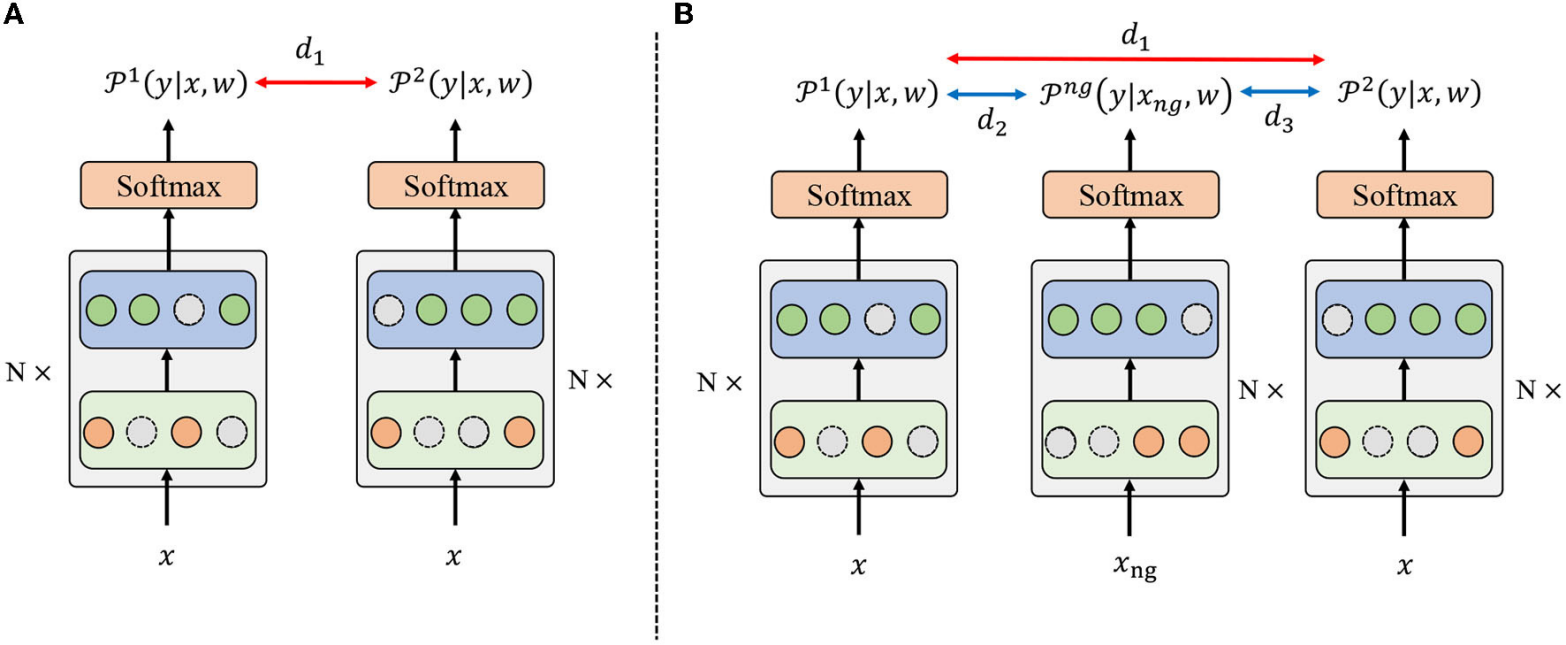
The overall framework for R-Drop and the proposed negative regularization enhanced R-Drop scheme. The backbone is based on Swin Transformer. **(A)** In R-Drop, the input data *x* goes through the model with a dropout mechanism twice, resulting in two different distributions, i.e., $P^{1}\left(y|x,w\right)$ and $P^{2}\left(y|x,w\right)$. *d*_1_ denotes the bidirectional KL divergence between $P^{1}\left(y|x,w\right)$ and $P^{2}\left(y|x,w\right)$. **(B)** R-Drop with negative regularization additionally introduces negative samples *x*_*ng*_ to obtain a distribution $P^{ng}\left(y|x,w\right)$. *d*_2_ and *d*_3_ represent the distance between the negative and two positive samples.

Based on the randomness introduced by the dropout mechanism, R-Drop (Wu et al., 2021) is proposed to regularize the output predictions of the model. Specifically, as shown in Figure 2A, R-Drop forces the output distributions of different sub-models generated by dropout to be consistent with each other, resulting in better performance and model generalization ability. However, R-Drop did not consider the difference between positive and negative samples, which prevents the model from learning diverse feature representations among different categories and impedes the model's generalization ability. In such cases, the model would become overly complex and memorize the observed data distribution, where overfitting may occur.

Negative sampling is an essential technique for preventing overfitting and enhancing performance (Zhou et al., 2016). Therefore, to address the drawback of R-Drop, we propose a negative regularization-enhanced R-Drop scheme that takes the negative instances into account. The overall framework of the proposed negative regularization-enhanced R-Drop scheme is demonstrated in Figure 2B. Specifically, in addition to making the positive samples *x*_*i*_ pass through the model twice like R-Drop [resulting in $P^{1}\left(y|x,w\right)$ and $P^{2}\left(y|x,w\right)$], our method will make the negative instances *x*_*ng*_ pass through the model to obtain a distribution $P^{ng}\left(y|x_{ng},w\right)$. Intuitively, the negative distribution $P^{ng}\left(y|x_{ng},w\right)$ should be different from two positive distributions $P^{1}\left(y|x,w\right)$ and $P^{2}\left(y|x,w\right)$, which could help the model learn more diverse feature representations and increase the model generalization ability.

At each training step, we aim to (1) minimize the bidirectional KL divergence between $P^{1}\left(y|x,w\right)$ and $P^{2}\left(y|x,w\right)$, (2) maximize the distance between $P^{ng}\left(y|x_{ng},w\right)$ and $P^{1,2}\left(y|x,w\right)$, and (3) match the predictive class to the actual class label. Specifically, for target (1), we use the same regularization objective as R-Drop with Equation 2. For target (2), we maximize the mean square error (MSE) between the negative and positive output distributions, which is:


(5) $$\begin{array}{l} L_{NG}={\left(\frac{1}{2}\left(P^{1}\left(y|x,w\right)+P^{2}\left(y|x,w\right)\right)-P^{ng}\left(y|x_{ng},w\right)\right)}^{2}. \end{array}$$

For target (3), we optimize the cross entropy learning objective $L_{CE}$ of the two forward passes $P^{1}\left(y|x,w\right)$ and $P^{2}\left(y|x,w\right)$:


(6) $$\begin{array}{l} L_{CE}=-y\log\left(P^{1}\right)-y\log\left(P^{2}\right). \end{array}$$

Hence, the final training objective is to optimize L for given data (*x, y*),


(7) $$\begin{array}{l} L=L_{CE}+\alpha \left(L_{KL}-L_{NG}\right), \end{array}$$

where α is the coefficient weight. In order to avoid the introduction of more hyperparameters, we only use α to control the weight of losses $L_{KL}$ and $L_{NG}$. Compared to the loss function of the R-Drop method, the proposed method additionally incorporates $L_{NG}$ into the learning process.

Recalling the definition for the test error of the full model, combined with the training objective in Equation 7, we have:


(8) $$\begin{array}{r} \begin{array}{r} R(f_{Full})\leq \underset{{ℒ}_{NG}\to \epsilon_{gb}}{\underset{︸}{\left|R(E_{\varepsilon }(f_{\varepsilon }))-R_{T}(E_{\varepsilon }(f_{\varepsilon }))\right|}}+\underset{{ℒ}_{CE}\to \epsilon_{te}}{\underset{︸}{R_{T}(E_{\varepsilon }(f_{\varepsilon }))}}+ \end{array}\\ \begin{array}{l} \underset{{ℒ}_{KL}\to \epsilon_{sf}}{\underset{︸}{\left|R(f_{Full})-R(E_{\varepsilon }(f_{\varepsilon }))\right|}}, \end{array} \end{array}$$

where the $L_{CE}$ is used to minimize the training error. Meanwhile, our method uses the $L_{KL}$ loss to alleviate the training-inference mismatch like R-Drop. Besides, our method also optimizes the positive and negative divergence of sub-models to reduce the generalization error of the sub-models with $L_{NG}$ loss. Intuitively, the proposed negative regularized dropout scheme could make the model learn more robust representations by considering diverse features from positive and negative samples. In this way, the proposed method regularizes the model space beyond dropout to maximize the distinction of different classification samples in the dataset.

### 4.3. Max-minus negative sampling

**Figure 3:**
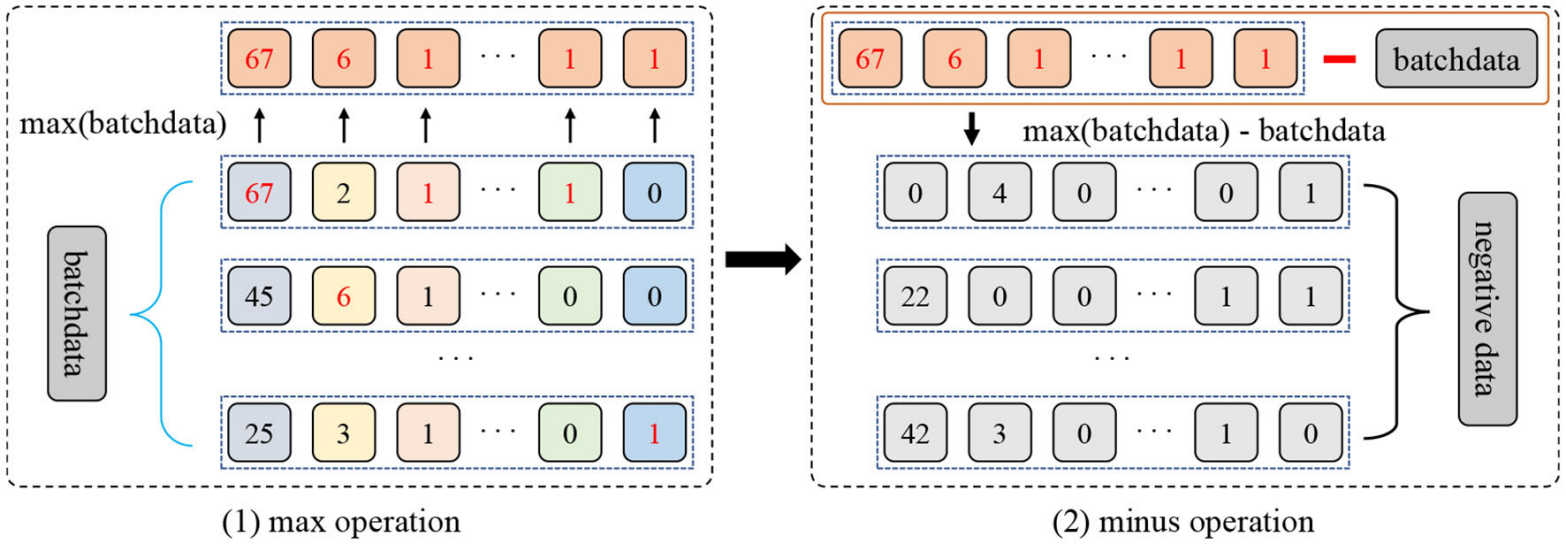
The sketch for the proposed max-minus negative sampling technique.

Typically, the different subsets of the original sample are considered positive samples, and the rest of the samples in the batch are considered negative samples. Unfortunately, such negative sampling does not bring sufficient diversity to the model, especially when it comes from the context of the same source domain. To reduce this issue, in this paper, we present a novel max-minus negative sampling strategy where the generated negative samples are unrelated to any category in the dataset. Generally, as shown in Figure 3, the procedure of the proposed max-minus negative sampling contains two stages. Concretely, the first stage is picking up the maximum values of all features in a mini-batch *x*_*b*_, i.e., max(*x*_*b*_). The second stage employs the collected maximum values minus the mini-batch to yield the negative samples *x*_*ng*_, i.e., *x*_*ng*_ = max(*x*_*b*_)−*x*_*b*_. In this way, the collected negative samples are quite different from the dataset samples. Then, the negative output distribution $P^{ng}\left(y|x_{ng},w\right)$ is obtained by feeding negative samples *x*_*ng*_ to the model with a dropout. In summary, the proposed max-minus negative sampling strategy produces more semantically dissimilar negative samples, ensuring sufficient diversity in the negative samples. For example, in our generated negative samples, after the max-minus operation, a patient who is 6 years old has a course of treatments lasting 5 years, which is irrational. Hence, these negative samples can help improve the model's robustness and avoid overfitting in the training and reasoning process.

### 4.4. Algorithm summary

In this paper, we utilize Swin Transformer as our feature extraction backbone. The overall framework of the proposed algorithm is presented in Algorithm 1. We initialize the parameters of Swin Transformer and prepare training data $D_{tr}={\left\{\left(x_{i},y_{i}\right)\right\}}_{i=1}^{n}$. At each training step, Lines 2–3 acquire the positive and negative data from $D_{tr}$. Lines 4–5 obtain the model output distributions $P^{1}\left(y_{i}|x_{i},w\right)$, $P^{2}\left(y_{i}|x_{i},w\right)$, and $P^{ng}\left(y_{i}|x_{ng},w\right)$. Lines 6–8 calculate the loss function, and Line 9 updates the model parameters.

**Algorithm 1:** The pseudocode of the proposed algorithm.

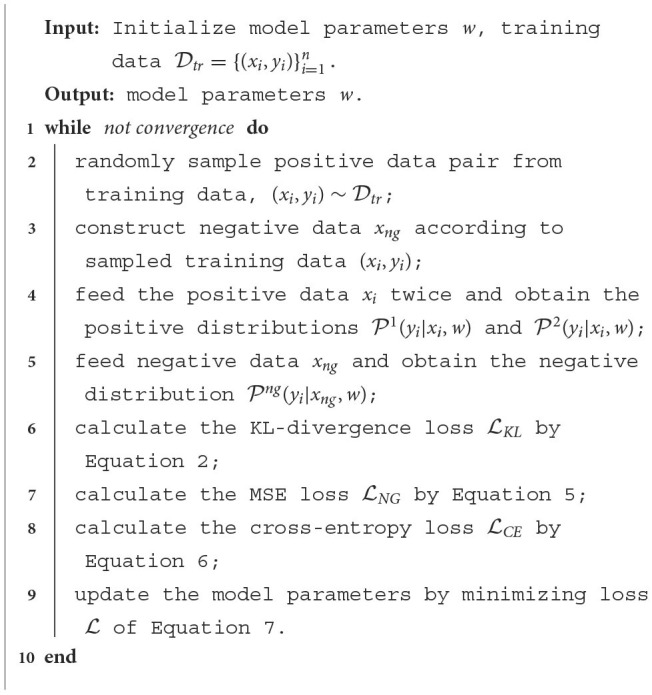

## 5. Experiments

### 5.1. Datasets description

Three medical prediction datasets were used in our experiments. The Pima Indian Diabetes (PID) dataset and the Wisconsin Breast Cancer (WBC) dataset were obtained from the UCI machine learning repository (ASUNCION, 2007), which are open-source databases. The third dataset (our dataset) was collected from the Chinese National Science and Technology Major Project TCM Syndrome Biological Technology Platform. The statistics of the three datasets are shown in Table 1. Note that WBC and PID datasets are complete, but our dataset contains 275 missing values. When making a medical diagnosis, in some circumstances, a conclusive diagnosis might not be achievable until more characteristics are obtained, especially for the traditional Chinese medicine treatment situation. Hence, for analysis purposes, any data with more than three missing values have been excluded from our dataset.

**Table 1:** The statistics of the three datasets.

**Statistics**	**WBC dataset**	**PID dataset**	**Our dataset**
No. of instances	683	768	1920
No. of categories	2	2	7
No. of features	9	8	24
No. of missing values	0	0	275
No. of training instances	478	537	1,344
No. of testing instances	205	231	576

Our dataset was collected from traditional Chinese medicine treatments for T2DM syndrome. The diabetes syndromes are divided into seven classes, including Qi and Yin deficiency with blood stasis, Qi and Yin deficiency, Qi and Yin deficiency with dampness, Qi deficiency and blood stasis, dampness and blood stasis, dampness and heat, and Qi stagnation and blood stasis. Our dataset consists of 24 features, where the age and course of treatment of the patient are continuous values, and the other features are boolean values. Note that only the boolean features contain incomplete data. Additionally, our dataset indicates that there are more male patients than female patients, with a higher number of patients in the age group of 40–80 years old and a course of 5–20 years. The primary symptoms of the disease include dry mouth, thirst, numbness, and tingling of the lower limbs. Note that our collected dataset does not include missing data for continuous values.

The WBC dataset includes extracted features from images, which comprises 683 data points, each with nine feature descriptions. The feature values range from 1 to 10, with 1 indicating a normal or benign case and 10 indicating the most abnormal case, based on the diagnosis. The Pima Indian Diabetes (PID) dataset comprises 768 data points, each with eight medical features. Of these, 268 data points correspond to diabetic patients, while 500 data points correspond to individuals without diabetes.

### 5.2. Comparison algorithm and training details

#### 5.2.1. Comparison algorithm

Our proposed algorithm utilizes the widely popular Swin Transformer (Liu et al., 2021) network as the model structure. In this paper, our experiments compare the following algorithm:

- Deep neural network (DNN): it is a type of neural network that has multiple layers allowing it to learn and represent complex patterns in data.
- ResNet50 architecture (He et al., 2016): it is a variant of the ResNet model which has 50 layers and utilizes residual connections to enable training of much deeper neural networks.
- Transformer (Vaswani et al., 2017): it is a neural network architecture that utilizes self-attention mechanisms to process sequential data, commonly used in natural language processing tasks such as language translation and text generation.
- Swin Transformer (Liu et al., 2021): it is a recent variant of the Transformer architecture that introduces hierarchical feature extraction and window-based self-attention to achieve state-of-the-art performance in computer vision tasks such as image classification and object detection.
- R-Drop (Wu et al., 2021): the R-Drop method aims to regularize the model predictions by minimizing the bidirectional KL divergence to improve the model's generalization. Note that the R-Drop method applied in this paper is also based on the Swin Transformer structure.

#### 5.2.2. Implementing and training details

We directly use the open-source implementation based on PyTorch^1^ for the comparison algorithms. DNN is a 5-layer deep learning model with 256 hidden-size layers. We use the open source for ResNet50^2^ and Transformer.^3^ In this paper, our proposed algorithm and R-Drop are implemented on the Swin Transformer.^4^ Additionally, we set the embedding size and hidden size to be the same for all methods for a fair comparison. For Swin Transformer, we configure the path size to 4, the window size to 7, the embedding size to 96, the model's depths to [2, 2, 4, 2], and the number of classification heads to [2, 2, 2, 4]. Each method runs five trials for obtaining the average performance.

#### 5.2.3. Evaluation metrics

As presented in Table 2, a confusion matrix shows how many predictions are correct and incorrect per class. For investigating the performance of the classification model, this paper used the following metrics:

- Accuracy: it measures the percentage of correctly classified instances out of all instances in the dataset, i.e., $Accuracy=\frac{TP+TN}{TP+TN+FP+FN}$.
- F1 score: it represents the harmonic means of precision and recall, i.e., $F1=\frac{2\times TP}{2\times TP+FP+FN}$.

**Table 2:** Confusion matrix.

	**True positive**	**True negative**
Predicted positive	*TP*	*FP*
Predicted negative	*FN*	*TN*

### 5.3. Main results

**Figure 4:**
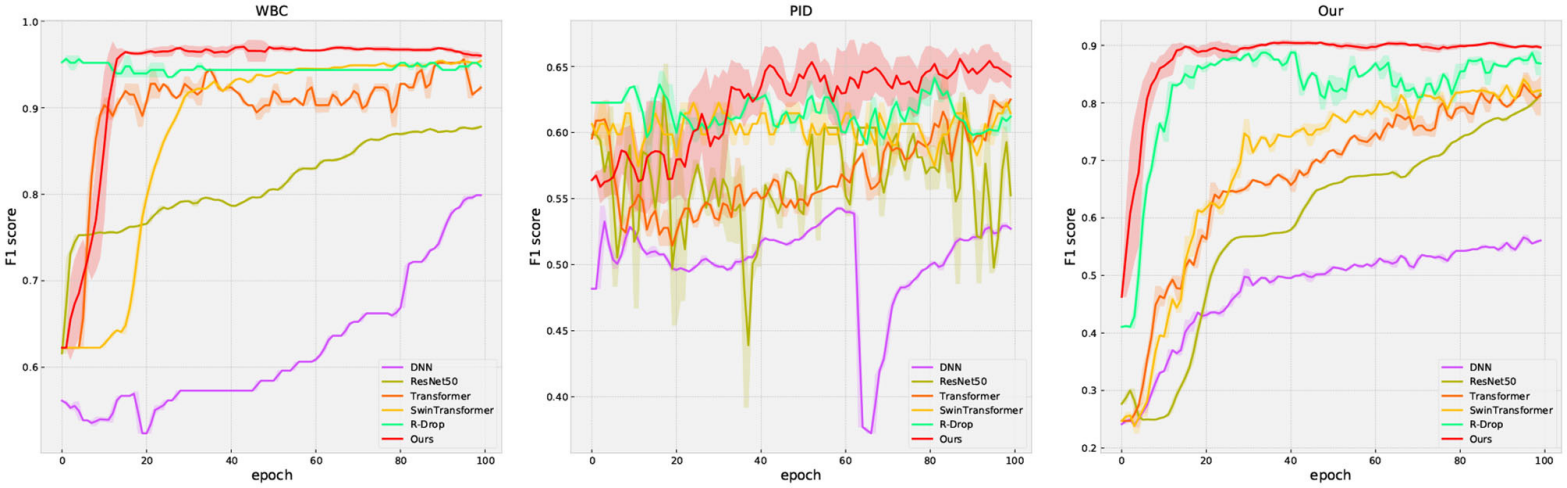
The evaluation curve of all methods on three datasets. The solid curves represent the mean of the evaluated data, while the shaded region indicates one standard deviation over five runs.

The performance comparison for all methods is presented in Table 3 and Figure 4. In Figure 4, the solid curve represents the averaged F1 score over five random trials, while the shaded region indicates one standard deviation. Additionally, the maximum average performance in Table 3 is the highest average value obtained from five trials. These results have the following suggestion: (1) our method outperforms or matches the baselines in terms of final performance across all datasets (including missing and complete datasets). For example, compared with the famous methods such as Swin Transformer, our method obtains 0.7392 (+3.81%) on accuracy and 0.6811 (+5.85%) on F1 score on the PID dataset, respectively; (2) regularization methods (R-Drop and ours) present better performance compared to other methods, which clearly shows the effectiveness of the regularization technique. Moreover, our method outperforms R-Drop (removing negative regularization, our method reduces to base R-Drop), which directly validates the effectiveness of the proposed negative regularization scheme; (3) our method exhibits a superior convergence rate compared to the baselines. Particularly, in our dataset, our method learns significantly faster than other methods; (4) our method achieves excellent model stability compared to other methods across all datasets, especially in our collected dataset. These results confirm that the proposed max-minus negative regularized dropout scheme improves the model generalization ability and maintains the training stability, particularly in the context of missing data.

**Table 3:** Performance comparison on three datasets.

**Methods**	**WBC**		**PID**		**Our dataset**	
	**Accuracy**	**F1 score**	**Accuracy**	**F1 score**	**Accuracy**	**F1 score**
DNN	0.8115	0.7989	0.6494	0.5443	0.6427	0.5703
ResNet50	0.8992	0.8788	0.7232	0.6517	0.8713	0.8244
Transformer	0.9715	0.9598	0.7013	0.6473	0.8819	0.8524
Swin Transformer	0.9633	0.9554	0.7013	0.6226	0.9019	0.8475
R-Drop	0.9781	0.9602	0.6897	0.6463	0.9349	0.8938
Our method	**0.9854**	**0.9762**	**0.7392**	**0.6811**	**0.9461**	**0.9101**

The highest metrics are bolded.

### 5.4. Ablation study

In this section, we conduct extensive ablation studies from different perspectives to gain a better understanding of the proposed method. The experiments are performed on the three datasets.

#### 5.4.1. Imputation for missing data

Missing data is a common occurrence in medical datasets for various reasons. We employed mode imputation, random imputation, Bayesian imputation, and NaN-replace imputation to fill in missing data with a boolean value (since our dataset didn't have missing data with continuous values). To investigate the impact of imputation techniques on performance, we tested all comparison methods with different imputed datasets. The experimental results are shown in Table 4. The mode imputation method achieves the best performance compared to other imputation techniques. Additionally, Bayesian imputation delivers a better F1 score result on ResNet50.

**Table 4:** Performance evaluation of different models with various imputation methods on our collected datatset.

	**DNN**	**ResNet50**	**Transformer**	**SwinTransformer**	**R-Drop**	**Ours**
**Imputation**	**F1 score**	**F1 score**	**F1 score**	**F1 score**	**F1 score**	**F1 score**
Mode	0.5703	0.8244	**0.8524**	**0.8475**	**0.8938**	**0.9101**
Random	**0.5844**	0.8053	0.8448	0.8281	0.8924	0.8938
Bayesian	0.4717	**0.8298**	0.8503	0.8299	0.8841	0.8524
NaN-replace	0.4690	0.8098	0.8349	0.8162	0.8467	0.8998

The NaN-replace imputation method means utilizing a default value to replace all missing values, such as −1. The best performance is highlighted in bold.

#### 5.4.2. Negative sampling techniques

**Figure 5:**
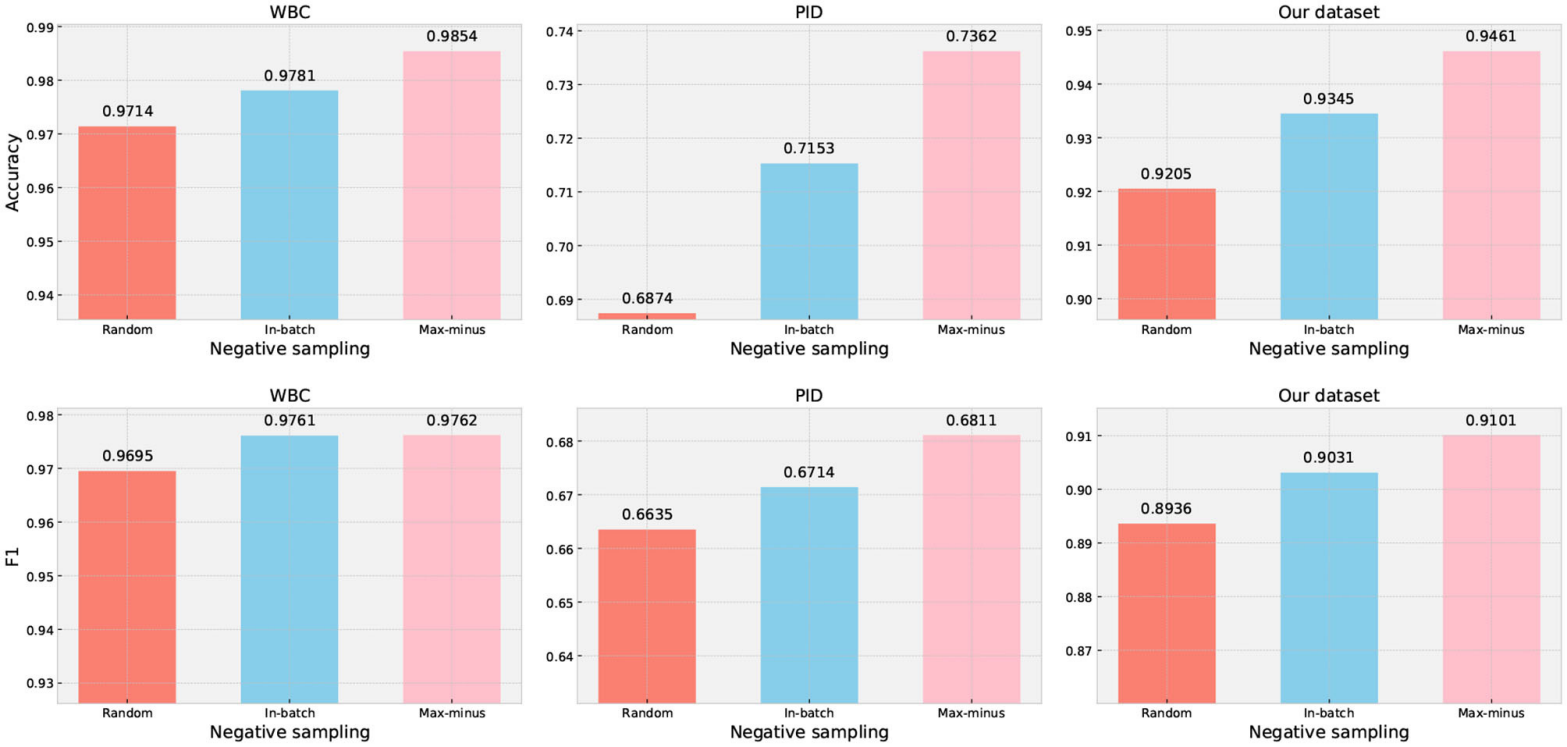
Performance evaluation of different negative sampling techniques on three datasets. Random generation means generating samples randomly within a given range, but the generated samples are not existing in the dataset. In-batch data is randomly choosing samples from the dataset that are not subject to the target class. Max-minus is the proposed negative sampling technique.

The negative sampling aims to provide the model with a wider range of examples to learn general patterns, which can enhance its ability to accurately differentiate between target and noise cases. In medical prediction data, different disease symptoms may exhibit subtle differences in clinical characteristics. Therefore, randomly selecting samples from the dataset may not be the most effective approach for improving the model's ability to capture subtle differences. We designed an experiment to evaluate the impact of different negative sampling techniques on classification performance based on our method. The results of our trial are presented in Figure 5, which clearly demonstrate that the proposed max-minus negative sampling technique delivers the best performance on all datasets compared to other negative sampling strategies. For example, the proposed max-minus negative sampling technique achieves an average improvement in accuracy by 3.49% and an F1 score by 1.37% on the PID dataset, respectively.

#### 5.4.3. Dropout rates

We study the performance of the proposed method with different dropout rates. Specifically, we set various dropout values to train our model. The experimental results are shown in Table 5. As we can see that with a wide range of dropout rates, our method consistently yields strong results. Even if the dropout rate is 0.5, meaning that half of the units are expected to be dropped randomly, our method can still achieve a satisfactory result (0.8524 F1 score) in comparison to the base Swin Transformer (0.8475 F1 score) on our dataset. These results confirm the effectiveness and robustness of our method.

**Table 5:** Performance evaluation of our method with different dropout rates.

	**WBC**		**PID**		**Our dataset**	
**Dropout rate**	**Accuracy**	**F1 score**	**Accuracy**	**F1 score**	**Accuracy**	**F1 score**
0.1	**0.9854**	0.9647	0.6935	0.6714	0.9349	0.9028
0.2	0.9781	0.9722	**0.7362**	**0.6811**	0.9323	0.9028
0.3	**0.9854**	**0.9762**	0.7338	0.6757	**0.9461**	**0.9101**
0.4	0.9781	0.9671	0.7232	0.6757	0.9297	0.8938
0.5	0.9528	0.9368	0.6802	0.6463	0.8933	0.8524

The best performance is highlighted in bold.

#### 5.4.4. Comparison of the KL loss and MSE loss

**Figure 6:**
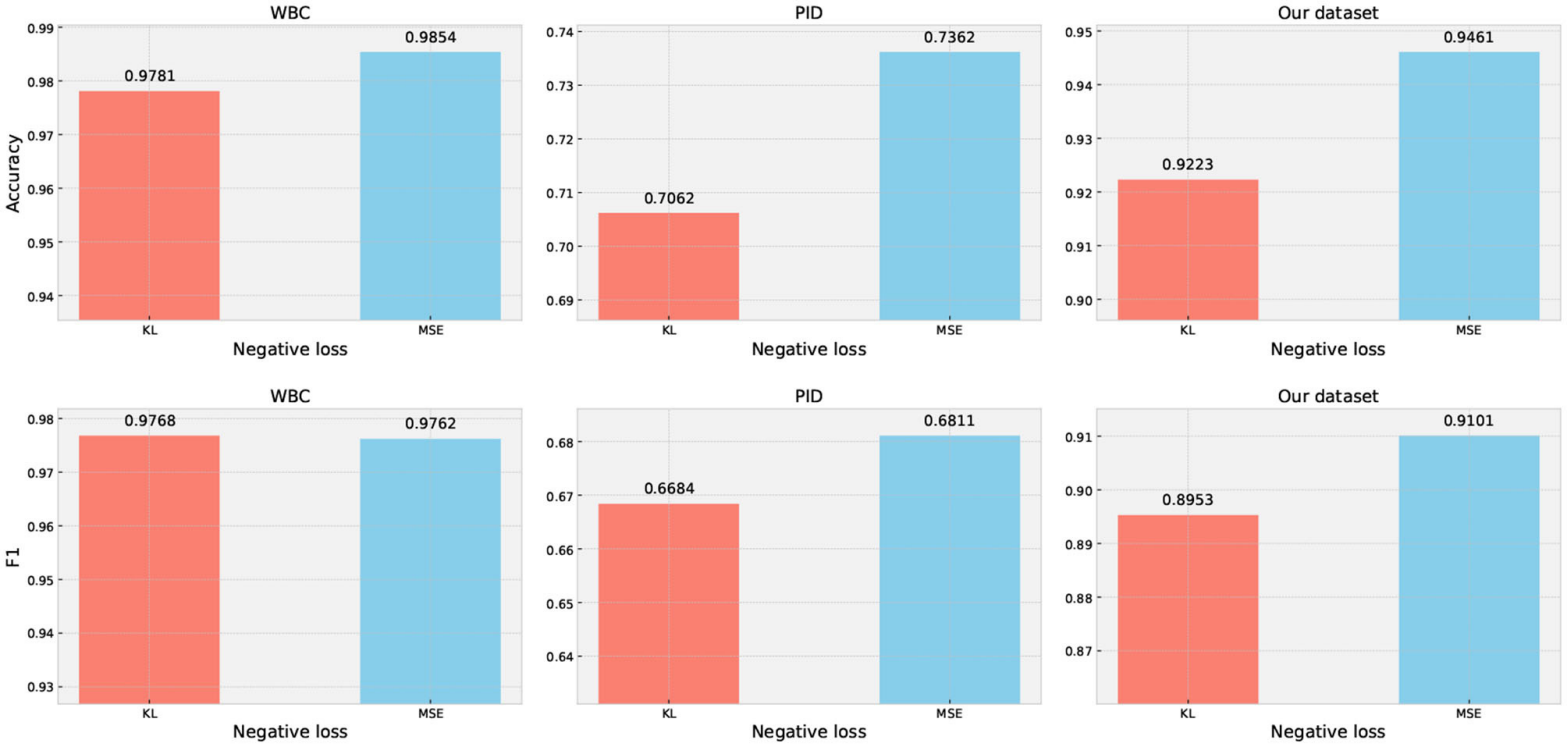
Performance evaluation of our method with different negative loss functions.

A well-designed loss function is essential for achieving good performance in machine learning tasks as it ensures that the model can learn meaningful patterns from the data and make accurate predictions. We designed an experiment to explore the impact of different loss functions for maximizing negative and positive output distributions on classification performance. Specifically, to enhance the model's ability to distinguish different categories' samples, we utilized bidirectional KL divergence loss and MSE loss to measure the negative and positive prediction distributions. The experimental results are demonstrated in Figure 6. Our results indicate that the MSE loss function outperforms the bidirectional KL divergence loss function in terms of the final performance. For instance, on PID, the MSE loss achieved a performance improvement in accuracy by 3% and F1 score by 1.27% compared to the bidirectional KL divergence loss function.

#### 5.4.5. Effect of weight α

Further, we explore the impact of the loss weight α by varying the weight α. As shown in Table 6, we have the following observation: (1) the best performance on each dataset is derived from different loss weight α, (2) larger α usually results in better performance compared to smaller ones. These findings mean that the choice of α varies across datasets. Different data distributions should use different α to regularize the model, depending on the specific data size for each task and how easily the model size can lead to overfitting.

**Table 6:** Performance evaluation of our method with different α.

**α**	**WBC**		**PID**		**Our dataset**	
	**Accuracy**	**F1 score**	**Accuracy**	**F1 score**	**Accuracy**	**F1 score**
0.001	0.9781	0.9647	0.6958	0.6729	0.8971	0.8852
0.01	**0.9854**	**0.9762**	0.7062	0.6782	0.9349	0.9013
0.1	0.9781	0.9518	0.7062	0.6684	0.9323	0.9163
1	0.9665	0.9437	0.7338	0.6757	0.9270	0.8938
2	0.9781	0.9647	**0.7362**	**0.6811**	**0.9461**	**0.9101**
4	0.9528	0.9368	0.7332	0.6757	0.9297	0.8938

The best performance is highlighted in bold.

## 6. Conclusion

In this paper, we propose a simple, yet effective negative regularization scheme built upon R-Drop to further boost performance and generalization ability, particularly in the context of missing data. The proposed negative regularization enhanced R-Drop scheme maintains both the consistency between two distributions from the same data sample and the inconsistency between the output distributions of positive and negative samples. Besides, we design a new max-minus negative sampling strategy, which facilitates convergence and is more effective compared to the traditional in-batch negative example sampling strategy. Extensive experimental results on three real-world medical datasets including both complete and missing data cases validate the effectiveness of the proposed method, particularly in the context of missing data.

## Data availability statement

The raw data supporting the conclusions of this article will be made available by the authors, without undue reservation.

## Author contributions

LH, CW, and YR conceived of the presented idea. XC conducted the experiments. LH wrote the manuscript with support from CW and YR. All authors discussed the results and contributed to the final manuscript.

## Footnotes

^1^ https://github.com/pytorch/pytorch

^2^ https://github.com/wangyunjeff/ResNet50-MNIST-pytorch

^3^ https://github.com/huggingface/transformers

^4^ https://github.com/microsoft/Swin-Transformer

## References

[B1] Amit Y. (2019). Deep learning with asymmetric connections and hebbian updates. Front. Comput. Neurosci. 13, 18. 10.3389/fncom.2019.00018 31019458 PMC6458299

[B2] ASUNCION A. (2007). UCI Machine Learning Repository. Available online at: https://archive.ics.uci.edu/datasets

[B3] Ayon S. I. Islam M. M. (2019). Diabetes prediction: a deep learning approach. Int. J. Inform. Eng. Electr. Bus. 12, 21. 10.5815/ijieeb.2019.02.0336091536

[B4] Batista G. E. Monard M. C. (2003). An analysis of four missing data treatment methods for supervised learning. Appl. Artif. Intell. 17, 519–533. 10.1080/713827181

[B5] Bell M. L. Fiero M. Horton N. J. Hsu C.-H. (2014). Handling missing data in rcts; a review of the top medical journals. BMC Med. Res. Methodol. 14, 1–8. 10.1186/1471-2288-14-118 25407057 PMC4247714

[B6] Bielza C. Larrañaga P. (2014). Bayesian networks in neuroscience: a survey. Front. Comput. Neurosci. 8, 131. 10.3389/fncom.2014.00131 25360109 PMC4199264

[B7] Donders A. R. T. Van Der Heijden G. J. Stijnen T. Moons K. G. (2006). A gentle introduction to imputation of missing values. J. Clin Epidemiol. 59, 1087–1091. 10.1016/j.jclinepi.2006.01.014 16980149

[B8] Enders C. K. Mistler S. A. Keller B. T. (2016). Multilevel multiple imputation: a review and evaluation of joint modeling and chained equations imputation. Psychol. Methods 21, 222. 10.1037/met0000063 26690775

[B9] Fan M. Yang A. C. Fuh J.-L. Chou C.-A. (2018). Topological pattern recognition of severe Alzheimer's disease via regularized supervised learning of EEG complexity. Front. Neurosci. 12, 685. 10.3389/fnins.2018.00685 30337850 PMC6180281

[B10] Gal Y. Ghahramani Z. (2016). “Dropout as a bayesian approximation: representing model uncertainty in deep learning,” in International Conference on Machine Learning. New York, NY: PMLR, 1050–1059.

[B11] Gao T. Yao X. Chen D. (2021). Simcse: simple contrastive learning of sentence embeddings. arXiv preprint arXiv:2104.08821. 10.18653/v1/2021.emnlp-main.55236568019

[B12] Ge S. Mishra S. Li C.-L. Wang H. Jacobs D. (2021). Robust contrastive learning using negative samples with diminished semantics. Adv. Neural Inform. Process. Syst. 34, 27356–27368. Available online at: https://proceedings.neurips.cc/paper_files/paper/2021/file/e5afb0f2dbc6d39b312d7406054cb4c6-Paper.pdf

[B13] Graham J. W. Cumsille P. E. Shevock A. E. (2013). Methods for Handling Missing Data, Handbook of Psychology, 2nd Edn. John Wiley and Sons, Ltd. 10.1002/0471264385.wei0204

[B14] He K. Zhang X. Ren S. Sun J. (2016). “Deep residual learning for image recognition,” in Proceedings of the IEEE Conference on Computer Vision and Pattern Recognition (Las Vegas, NV), 770–778.

[B15] Jakobsen J. C. Gluud C. Wetterslev J. Winkel P. (2017). When and how should multiple imputation be used for handling missing data in randomised clinical trials–a practical guide with flowcharts. BMC Med. Res. Methodol. 17, 1–10. 10.1186/s12874-017-0442-1 29207961 PMC5717805

[B16] King G. Honaker J. Joseph A. Scheve K. (1998). “List-wise deletion is evil: what to do about missing data in political science,” in Annual Meeting of the American Political Science Association, Boston, Volume 52. (Washington, DC).

[B17] Kumar N. Hoque M. Shahjaman M. Islam S. Mollah M. Haque N.. (2017). Metabolomic biomarker identification in presence of outliers and missing values. BioMed Res. Int. 2017, 2437608. 10.1155/2017/2437608 28293630 PMC5331169

[B18] Li F. Fu M. Chen W. Zhang F. Zhang H. Qu H.. (2022). Improving exploration in actor–critic with weakly pessimistic value estimation and optimistic policy optimization. IEEE Trans. Neural Netw. Learn. Syst. 2022, 1–14. 10.1109/TNNLS.2022.3215596 36306289

[B19] Li Y. Gal Y. (2017). “Dropout inference in bayesian neural networks with alpha-divergences,” in International Conference on Machine Learning. Sydney, NSW: PMLR, 2052–2061.

[B20] Liu Z. Lin Y. Cao Y. Hu H. Wei Y. Zhang Z.. (2021). “Swin transformer: hierarchical vision transformer using shifted windows,” in Proceedings of the IEEE/CVF International Conference on Computer Vision (Montreal, BC), 10012–10022.

[B21] Loshchilov I. Hutter F. (2019). “Decoupled weight decay regularization,” in International Conference on Learning Representations (New Orleans, LA).

[B22] Ma Z. Chen G. (2018). Bayesian methods for dealing with missing data problems. Jo. Kor. Stat. Soc. 47, 297–313. 10.1016/j.jkss.2018.03.002

[B23] Mazumder R. Hastie T. Tibshirani R. (2010). Spectral regularization algorithms for learning large incomplete matrices. J. Machine Learn. Res. 11, 2287–2322. Available online at: https://www.jmlr.org/papers/volume11/mazumder10a/mazumder10a.pdf 21552465 PMC3087301

[B24] Mehrabani-Zeinabad K. Doostfatemeh M. Ayatollahi S. M. T. (2020). An efficient and effective model to handle missing data in classification. BioMed Res. Int. 2020, 8810143. 10.1155/2020/8810143 33299878 PMC7710403

[B25] Miller M. W. Sperbeck E. Robinson M. E. Sadeh N. Wolf E. J. Hayes J. P.. (2016). 5-HT2A gene variants moderate the association between ptsd and reduced default mode network connectivity. Front. Neurosci. 10, 299. 10.3389/fnins.2016.00299 27445670 PMC4923242

[B26] Miotto R. Wang F. Wang S. Jiang X. Dudley J. T. (2018). Deep learning for healthcare: review, opportunities and challenges. Brief. Bioinformat. 19, 1236–1246. 10.1093/bib/bbx044 28481991 PMC6455466

[B27] Moradi R. Berangi R. Minaei B. (2020). A survey of regularization strategies for deep models. Artif. Intell. Rev. 53, 3947–3986. 10.1007/s10462-019-09784-7

[B28] Musil C. M. Warner C. B. Yobas P. K. Jones S. L. (2002). A comparison of imputation techniques for handling missing data. West. J. Nurs. Res. 24, 815–829. 10.1177/019394502762477004 12428897

[B29] Pham H. Le Q. (2021). “Autodropout: learning dropout patterns to regularize deep networks,” in Proceedings of the AAAI Conference on Artificial Intelligence, Volume 35 (Vancouver, BC), 9351–9359.

[B30] Schafer J. L. Graham J. W. (2002). Missing data: our view of the state of the art. Psychol. Methods 7, 147. 10.1037/1082-989X.7.2.147 12090408

[B31] Schneider T. (2001). Analysis of incomplete climate data: estimation of mean values and covariance matrices and imputation of missing values. J. Clim. 14, 853–871. 10.1175/1520-0442014<0853:AOICDE>2.0.CO;2

[B32] Schroff F. Kalenichenko D. Philbin J. (2015). “FaceNet: a unified embedding for face recognition and clustering,” in Proceedings of the IEEE Conference on Computer Vision and Pattern Recognition (Boston, MA), 815–823.

[B33] Spoon K. Tsai H. Chen A. Rasch M. J. Ambrogio S. Mackin C.. (2021). Toward software-equivalent accuracy on transformer-based deep neural networks with analog memory devices. Front. Comput. Neurosci. 15, 675741. 10.3389/fncom.2021.675741 34290595 PMC8287521

[B34] Srivastava N. Hinton G. Krizhevsky A. Sutskever I. Salakhutdinov R. (2014). Dropout: a simple way to prevent neural networks from overfitting. J. Machine Learn. Res. 15, 1929–1958. Available online at: https://jmlr.org/papers/volume15/srivastava14a/srivastava14a.pdf33259321

[B35] Teipel S. J. Grothe M. J. Metzger C. D. Grimmer T. Sorg C. Ewers M.. (2017). Robust detection of impaired resting state functional connectivity networks in Alzheimer's disease using elastic net regularized regression. Front. Aging Neurosci. 8, 318. 10.3389/fnagi.2016.00318 28101051 PMC5209379

[B36] Templ M. Kowarik A. Filzmoser P. (2011). Iterative stepwise regression imputation using standard and robust methods. Comput. Stat. Data Anal. 55, 2793–2806. 10.1016/j.csda.2011.04.012

[B37] Thirukumaran S. Sumathi A. (2012). “Missing value imputation techniques depth survey and an imputation algorithm to improve the efficiency of imputation,” in 2012 Fourth International Conference on Advanced Computing (ICoAC). (Chennai: IEEE), 1–5.

[B38] Vaswani A. Shazeer N. Parmar N. Uszkoreit J. Jones L. Gomez A. N.. (2017). Attention is all you need. Adv. Neural Inform. Process. Syst. 30, 1–11. Available online at: https://proceedings.neurips.cc/paper_files/paper/2017/file/3f5ee243547dee91fbd053c1c4a845aa-Paper.pdf

[B39] Waljee A. K. Mukherjee A. Singal A. G. Zhang Y. Warren J. Balis U.. (2013). Comparison of imputation methods for missing laboratory data in medicine. Br. Med. J. Open 3, e002847. 10.1136/bmjopen-2013-002847 23906948 PMC3733317

[B40] Wang S. Chen Y. Zhuo J. Huang Q. Tian Q. (2018). “Joint global and co-attentive representation learning for image-sentence retrieval,” in Proceedings of the 26th ACM International Conference on Multimedia (Seoul), 1398–1406.

[B41] Wong D. D. Fuglsang S. A. Hjortkjær J. Ceolini E. Slaney M. De Cheveigne A. (2018). A comparison of regularization methods in forward and backward models for auditory attention decoding. Front Neurosci. 12, 531. 10.3389/fnins.2018.00531 30131670 PMC6090837

[B42] Wu L. Li J. Wang Y. Meng Q. Qin T. Chen W.. (2021). R-drop: regularized dropout for neural networks. Adv. Neural Inform. Process. Syst. 34, 10890–10905. Available online at: https://openreview.net/pdf?id=bw5Arp3O3eY

[B43] Yang Z. Qu H. Fu M. Hu W. Zhao Y. (2023). A maximum divergence approach to optimal policy in deep reinforcement learning. IEEE Trans. Cybernet. 53, 1499–1510. 10.1109/TCYB.2021.3104612 34478393

[B44] Zhang H. Wang B. Chen C. Sun Y. Chen J. Tan X.. (2022). Sleep patterns, genetic susceptibility, and incident chronic kidney disease: a prospective study of 370,671 participants. Front. Neurosci. 16, 44. 10.3389/fnins.2022.725478 35173575 PMC8843034

[B45] Zhou B. Khosla A. Lapedriza A. Oliva A. Torralba A. (2016). “Learning deep features for discriminative localization,” in Proceedings of the IEEE Conference on Computer Vision and Pattern Recognition (Las Vegas, NV), 2921–2929.

